# Multiple pro-tumor roles for protein acyltransferase DHHC3

**DOI:** 10.18632/oncoscience.385

**Published:** 2017-12-20

**Authors:** Chandan Sharma, Martin E. Hemler

**Affiliations:** Dana-Farber Cancer Institute, Boston, MA, USA

**Keywords:** DHHC3, oxidative stress, senescence, TXNIP, immune activation

## DHHC3 AND CANCER

Members of the ‘DHHC’ (Asp-His-His-Cys) family of 23 mammalian protein acyltransferases, traditionally studied in non-cancer contexts, have emerged as key regulators of tumor biology [1]. For example, melanomagenesis is negatively regulated by DHHC13 [2], and DHHC5 drives malignant glioma development and progression [3]. Recent evidence now also establishes Golgi-resident protein acyl transferase DHHC3 (GODZ) as a key cancer regulator [4].

Elevated DHHC3 expression was evident in malignant and metastatic breast cancer and in nearly all breast cancer subtypes, and analysis of TCGA data showed that *zDHHC3* upregulation correlates with significantly diminished overall survival of patients with breast cancer [4]. Furthermore, ablation of *zDHHC3* from human breast cancer cells caused a marked reduction in the sizes of both primary tumors and metastatic colonies in xenograft models [4]. DHHC3 levels are elevated not only in breast cancer, but also in colon and prostate cancer and elevated *zDHHC3* expression correlated with diminished survival in patients with six other cancers besides breast cancer [4]. Also, *zDHHC3* ablation led to markedly diminished prostate cancer growth in a xenograft model (our unpublished results). Together these results point to DHHC3 having a strong pro-tumor role in multiple cancers.

## MECHANISTIC INSIGHTS

*In vitro* tumor cell growth and morphology was minimally affected by *zDHHC3* ablation. However, *zDHHC3*-ablated tumor cells showed abundant evidence for upregulated oxidative stress, as seen by colorimetric assays, inhibition of phosphatase activity, increased TXNIP expression, and characteristic changes in expression levels of >10 different genes diagnostic for oxidative stress. Mass spectrometry was subsequently used to identify DHHC3 substrates, and this yielded several antioxidant-type proteins (manuscript in preparation). These results are again consistent with *zDHHC3* ablation causing elevated oxidative stress. Also, tumor cell senescence was elevated, as seen by a colorimetric assay, changes in expression of >29 senescence-related genes, and production of chemokines typical of a Senescence-Associated Secretory Phenotype (SASP) response. The SASP response was accompanied by recruitment of anti-tumor innate immune cells (e.g. NK cells, M1-like macrophages) into tumor sites. Notably, reconstitution of *zDHHC3*-ablated tumor cells with wild type DHHC3 (but not DHHC3 mutated at the active site needed for palmitoylation) reversed effects of *zDHHC3* ablation on TXNIP expression, chemokine production, oxidative stress, senescence and *in vivo* tumor growth. Hence, tumor-regulatory effects of DHHC3 clearly depend on its activity as a palmitoyl transferase.

**Figure 1:**
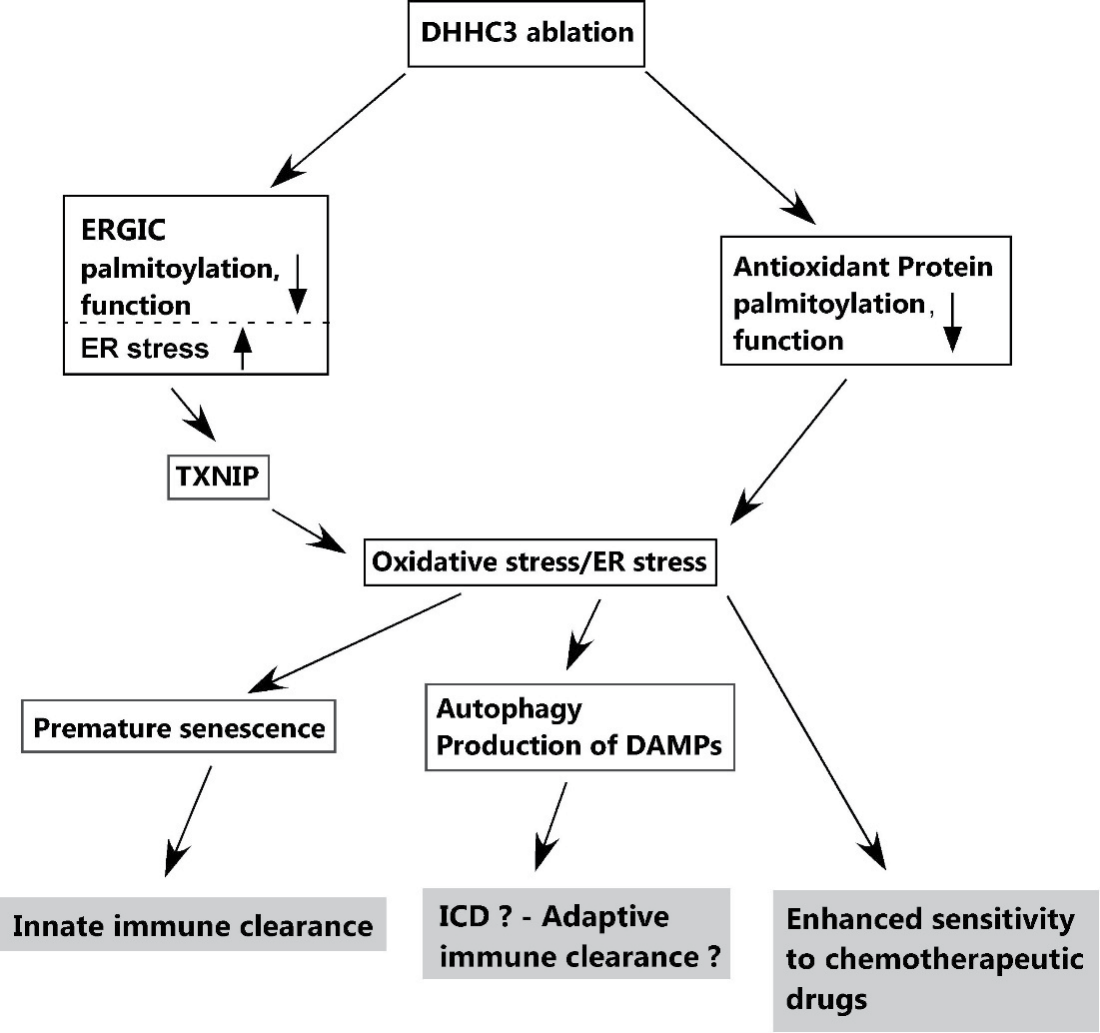
Actual and predicted consequences of ZDHHC3 ablation in tumor cells

The schematic in Fig. 1 emphasizes the effects of *zDHHC3* ablation leading to loss of anti-oxidant protections and elevated oxidative stress, which is partly dependent on upregulation of pro-oxidative stress protein TXNIP. To link *zDHHC3* ablation to upregulation of TXNIP (which is upregulated by ER stress), we established that ERGIC3 (endoplasmic reticulum Golgi intermediate compartment 3) protein, which inhibits ER stress, is also a substrate of DHHC3. Hence, ZDHHC3 ablation disrupted the palmitoylation, distribution, and presumably also ER stress-inhibiting function of ERGIC3, which at least partly explains upregulation of TXNIP (4). Elevated oxidative stress is known to trigger senescence, leading to recruitment of innate immune cells and tumor clearance (Fig. 1). These findings are novel because neither DHHC3 nor other enzymes in the DHHC enzyme family were previously linked to oxidative stress, ER stress, senescence, or immune system activation.

## OTHER ROLES FOR DHHC3

We predict that DHHC3 ablation may also affect adaptive immunity, perhaps through an immunogenic cell death (ICD) response (Fig. 1). Drug-induced ICD involves tumor cell release of danger signals (damage-associated molecular patterns; DAMPs), followed by recruitment and activation of dendritic cells, leading to mobilization of tumor-infiltrating lymphocytes. This process can provide a major boost to cancer therapy [5]. Consistent with this, ablation of *zDHHC3* triggered multiple hallmarks of ICD, including oxidative stress, ER stress, autophagy, EIF2A phosphorylation and production of DAMPs and (our unpublished results).

Furthermore, our unpublished results also indicate that *zDHHC3* ablation enhances tumor cell sensitivity to multiple different chemotherapeutic agents. This likely occurs because i) antioxidant proteins within cancer cells help to protect them from chemotherapeutic agents [6], and ii) *zDHHC3* ablation disables palmitoylation and proper function of several antioxidant proteins (Fig. 1).

## DHHC3 AS A POTENTIAL CANCER THERAPEUTIC TARGET

Because DHHC3 is upregulated in several cancers, correlates with diminished survival, directly affects tumor growth *in vivo*, and may inhibit both innate and adaptive anti-tumor immunity, we suggest that it may be an excellent therapeutic target. In this regard, enzymes are typically amenable to small molecule inhibition, and indeed, progress is being made in finding inhibitors for DHHC-type proteins [7]. Also, normal physiology is minimally altered in *zDHHC3* knockout mice [8], suggesting that it may be feasible to target DHHC3 in pathological settings (e.g. in cancer cells) without disrupting normal cell functions.
